# Risk of complete atypical femur fracture with Oral bisphosphonate exposure beyond three years

**DOI:** 10.1186/s12891-020-03672-w

**Published:** 2020-12-03

**Authors:** Joan C. Lo, Romain S. Neugebauer, Bruce Ettinger, Malini Chandra, Rita L. Hui, Susan M. Ott, Christopher D. Grimsrud, Monika A. Izano

**Affiliations:** 1 Division of Research, Kaiser Permanente Northern California, 2000 Broadway, Oakland, CA 94612 USA; 2 The Permanente Medical Group, Oakland, CA USA; 3 Pharmacy Outcomes Research Group, Kaiser Permanente California, Oakland, CA USA; 4 Department of Medicine, University of Washington School of Medicine, Seattle, WA USA; 5 Department of Orthopedic Surgery, Kaiser Permanente Oakland Medical Center, Oakland, CA USA

**Keywords:** Femur fracture, Subtrochanter, Femoral shaft, Atypical fracture, Bisphosphonates

## Abstract

**Background:**

Bisphosphonate (BP) therapy has been associated with atypical femur fracture (AFF). However, the threshold of treatment duration leading to increased AFF risk is unclear. In a retrospective cohort of older women initiating BP, we compared the AFF risk associated with treatment for at least three years to the risk associated with treatment less than three years.

**Methods:**

We used observational data from a large population of female members of an integrated healthcare system who initiated oral BP during 2002–2014. Women were retrospectively followed for incident AFF confirmed by radiologic adjudication. Demographic data, pharmacologic exposures, comorbidity, bone density, and fracture history were ascertained from electronic health records. Inverse probability weighting was used to estimate risk differences comparing the cumulative incidence (risk) of AFF if women discontinued BP within three years to the cumulative incidence of AFF if women continued BP for three or more years, adjusting for potential time-dependent confounding by the aforementioned factors.

**Results:**

Among 87,820 women age 45–84 years who initiated BP (mean age 68.6, median T-score − 2.6, 14% with prior major osteoporotic fracture), 16,180 continued BP for three or more years. Forty-six confirmed AFFs occurred during follow-up in the two groups. AFF-free survival was greater for BP treatment < 3 years compared to treatment ≥3 years (*p* = 0.004 comparing areas under survival curves). At five years, the risk of AFF was 27 per 100,000 (95% confidence interval, CI: 8–46) if women received BP treatment < 3 years and 120 per 100,000 (95% CI: 56–183) if women received BP treatment ≥3 years (risk difference 93 per 100,000, 95% CI: 30–160). By ten years, the risks were 27 (95% CI: 8–46) and 363 (95% CI: 132–593) per 100,000 for BP treatment < 3 and ≥ 3 years, respectively (risk difference 336 per 100,000, 95% CI: 110–570).

**Conclusions:**

Bisphosphonate treatment for 3 or more years was associated with greater risk of AFF than treatment for less than 3 years. Although AFFs are uncommon among BP-treated women, this increased risk should be considered when counseling women about long-term BP use. Future studies should further characterize the dose-response relationship between BP duration and incident AFF and identify patients at highest risk.

## Background

Atypical femur fractures (AFF) are an uncommon complication of oral bisphosphonate (BP) therapy [1], one of the first line therapies for osteoporosis and fracture prevention. These distinctive fractures do not appear to be related to osteoporotic bone fragility and may happen in the absence of a fall or apparent trauma. Following early case reports of non-traumatic subtrochanteric and femoral shaft fractures observed in postmenopausal women with prolonged BP exposure [2, 3], the definition of AFF evolved to encompass specific radiographic imaging criteria that include a primarily transverse fracture occurring in the femoral diaphysis, with minimal or no comminution, and the presence of localized periosteal or endosteal thickening of the lateral cortex (where these fractures originate) [1, 4, 5].

Duration of BP therapy appears to be a key determinant of AFF risk. In a large southern California population, Dell et al. [4] found that the age-adjusted incidence of AFF increased progressively over 8–10 years of BP exposure. We also found a progressive increase in AFF incidence through 8–10 years of BP exposure [6]. These two studies provide compelling evidence for a differential risk of AFF among short and long-term BP users, but did not adjust for gaps in BP treatment, the severity of osteoporosis, and other potential confounders [7]. A nationwide retrospective case-control study in Sweden examined 172 patients who experienced an AFF and 952 controls and reported a 2.5-fold increase in the adjusted odds of AFF for each additional year of BP use [8]. On the other hand, a post-hoc analysis of three randomized placebo-controlled clinical trials of women exposed to BP therapy for 3–5 and up to 10 years did not observe an association of BP treatment with diaphyseal femur fracture risk [9]; however of 14,195 subjects, only 3600 had been exposed to BP longer than 3 years and the radiographs in most cases were not examined [9]. In the absence of randomized trials designed to examine AFF risk, which are not possible due to the size and length of time required to study this adverse outcome, the question remains as to the time-point during BP treatment where AFF risk increases.

To address this question, we used observational data from a large existing cohort of women who initiated BP therapy [6] to examine the risk of AFF if women continued BP for three or more years in comparison to the risk of AFF if women discontinued treatment before 3 years. Recognizing that women at higher risk for fracture may receive longer BP treatment which may increase the risk of AFF, we used causal inference analytic methods [10, 11] to account for potential time-dependent confounding by factors related to both BP continuation and AFF risk.

## Methods

### Setting

Kaiser Permanente Northern California (KPNC) is a large integrated healthcare delivery system that serves over four million members. Since 1995, centralized electronic databases comprising pharmacy records, ambulatory visit and hospitalization diagnoses, clinical encounters, and imaging reports have been maintained, with the ability to link across administrative databases and membership records. Digital radiologic images have been centrally available starting in 2002, with such images from all KPNC imaging centers accessible by 2005.

### Study population

The study population included an existing cohort of all KPNC female members aged 45–84 years old who were identified from health plan pharmacy records as having initiated oral BP therapy with alendronate, risedronate or ibandronate between January 2002 and September 2014 [6]. Women without health plan membership for the 2 years before BP initiation and those who received intravenous BP (zoledronic acid, pamidronate or ibandronate) or etidronate any time before oral BP initiation were excluded. We also excluded women with any of the following within 2 years before BP initiation: diagnosed metastatic cancer beyond lymph nodes (ICD-9 197.x-199.0), multiple myeloma (ICD-9 203.0x), Paget’s disease of the bone (ICD-9 731.0), osteogenesis imperfecta (ICD-9 756.51), hypophosphatasia (ICD-9 275.3), and primary hyperparathyroidism (ICD-9 252.01); receipt of teriparatide or denosumab; advanced kidney disease defined by outpatient estimated glomerular filtration rate (eGFR) < 30 mL/min/1.73 m^2^ calculated using the Chronic Kidney Disease Epidemiology Collaboration (CKD-EPI) equation [12] or by prior receipt of chronic dialysis or renal transplantation. The index date was defined as the date the initial BP prescription was dispensed.

### Bisphosphonate (BP) exposure assessment

Bisphosphonate exposure was determined based on dispensing dates and days’ supply of each prescription. Stockpiling was allowed for prescriptions that overlapped 30 days or less; the second prescription took precedence when prescriptions overlapped for more than 30 days [13]. Each patient’s BP use was updated at each successive quarter (90-day interval) of follow-up after the index date. Women were categorized as “on BP” if at least half of the 90-day period was covered by a prescription (i.e., proportion of days covered (PDC) 0.50 or greater) [14]; otherwise women were considered “off BP”. Because our goal was to examine adverse outcomes associated with treatment rather than its efficacy (a measure potentially requiring a higher adherence threshold), we chose a 50% PDC adherence threshold to define exposure for this study. Classification of BP exposure regimens is described in *Statistical Analyses.*

### Follow-up and atypical femur fracture (AFF) assessment

Women were followed from the time of initial BP prescription until they: (a) experienced a complete AFF [6], (b) died, (c) developed an exclusionary condition as defined above, (d) ended health plan membership, or (f) reached 10 years of follow-up or end of study on 9/30/2015, whichever came first.

As previously described for the source cohort [6], potential AFF cases were identified from principal hospital discharge diagnoses for fractures of the femoral subtrochanter (ICD-9 820.22) and shaft (821.0x), pathologic fracture of the femur specified as other and not neck (ICD-9 733.15) and stress fracture of the femur (ICD-9 733.97), where case review was expanded to include principal diagnoses of open fracture codes (820.23 and 821.1x) and (per) trochanteric fractures (820.20, 820.21) when associated with a secondary diagnosis of diaphyseal, pathologic, or stress fracture accompanied by evidence (radiologic report or image) of diaphyseal fracture location. After excluding periprosthetic fractures, pathologic fractures, and fractures occurring outside the femoral diaphysis, radiologic images of diaphyseal fractures were adjudicated by a board-certified orthopedic surgeon with experience in identifying atypical femur fractures (blinded to BP treatment duration) [6].

**Fig. 1:**
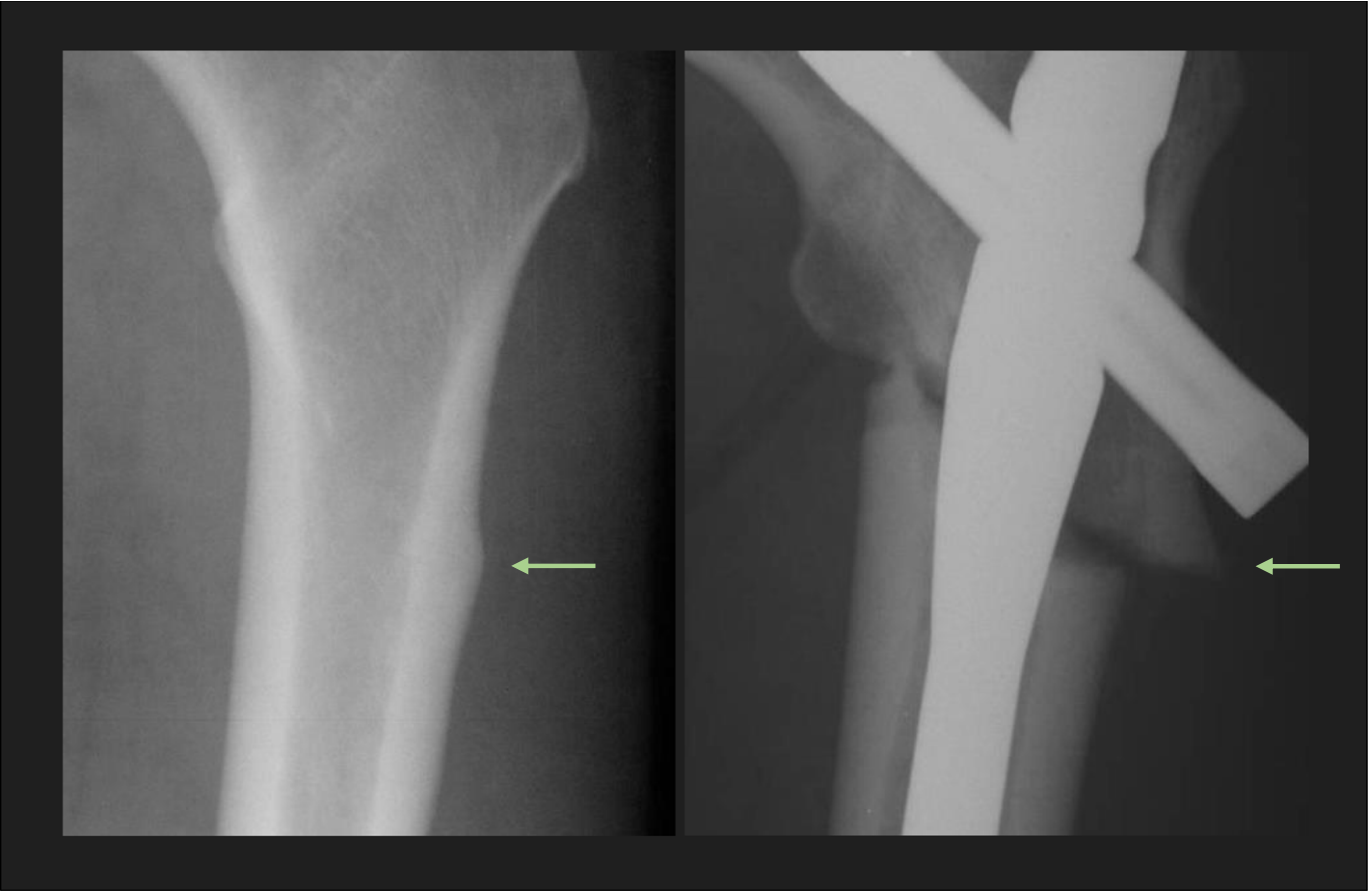
Example of an atypical femur fracture occurring at the site of focal cortical hypertrophy (arrow) [22] © Susan Ott, MD

The major criteria for AFF identified in the American Society for Bone and Mineral Research (ASBMR) Task Force 2013 Revised Case Definition of AFF [1] were used to classify complete diaphyseal fractures (fracture line extending through both cortices) and included the following required radiographic and clinical features: (1) noncomminuted or minimally comminuted fracture [4]; (2) fracture originating at the lateral cortex and primarily transverse in pattern (with or without a medial spike) [4, 5]; (3) evidence of localized periosteal or endosteal thickening of the lateral cortex at the site of fracture origin [1, 4, 15]; and (4) fracture occurring with minimal to no trauma. These radiographic features were first reported more than 10 years ago [3, 16, 17] and comprise the well-established criteria for AFF used by experts, especially the finding of focal periosteal or endosteal thickening at the transverse fracture origin [4, 18, 19]. The original ASBMR criteria for AFF [20] were revised in 2013 [1] to include periosteal callous formation as a major feature of AFF, with recognition that the initially transverse fracture originating in the lateral cortex can propagate medially at an oblique angle, with or without a medial spike [1, 15, 21]. It has also been noted that these atypical fractures can present initially with focal cortical hypertrophy that progresses to partial or complete AFF [1, 15, 16, 22]. Figure 1 shows a radiographic example of complete AFF [22], demonstrating the transverse fracture that develops in the lateral cortex at the site of periosteal hypertrophy, with the fracture becoming oblique as it propagates medially.

### Covariates

Electronic health record databases were used to derive covariate data for this study. Age was determined at index date and self-reported race-ethnicity was classified as non-Hispanic White, Black, Asian/Pacific Islander, Hispanic, and other or unknown. Neighborhood educational attainment and household income were estimated using 2010 US Census block data; residence in a census block with more than 25% of adults over age 25 years reporting below 12th grade education was used as a proxy for low educational attainment and residence in a census block with median household income <$35,000 was used as a proxy for low income. Patient index year (i.e. year of cohort entry) was classified as before 2008 or 2008 and later. We also ascertained the following baseline variables using the closest data available within 5 years before or after BP initiation: self-reported smoking status; body mass index (BMI) classified as normal or underweight (< 25 kg/m^2^), overweight (25 to < 30 kg/m^2^), or obese (≥30 kg/m^2^); and low vitamin D level (25OHD < 20 ng/mL), as well as an indicator of whether vitamin D levels were assessed. We did not have information on non-prescription cholecalciferol (Vitamin D3) supplementation, the most common approach for optimizing Vitamin D levels in patients with osteoporosis, including those with vitamin D deficiency who receive initial pharmacologic ergocalciferol therapy.

Time-dependent covariates, assessed at baseline and updated every 90 days, included: (1) the Charlson-Deyo Comorbidity Index (CCI) [23] derived from diagnostic and procedure codes associated with health plan encounters in the prior year (assessed annually) and categorized as 0, 1, 2, 3 or more; (2) diabetes mellitus, defined by having at least two clinical diagnoses and pharmacologic treatment; (3) rheumatoid arthritis, defined by at least two diagnoses; (4) grade 3A (eGFR 45–59 mL/min/1.7m^2^) and 3B (eGFR 30–44 mL/min/1.7m^2^) chronic kidney disease (CKD), using the most recent outpatient serum creatinine level [12, 24] (the mean eGFR of 74 mL/min/1.7m^2^ was used to impute baseline eGFR when missing, and an indicator of whether the value was imputed [25, 26] was used in analyses); (5) receipt of proton-pump inhibitors, aromatase inhibitors, estrogen, or raloxifene from pharmacy databases; (6) recent oral glucocorticoid exposure defined as receiving a cumulative prednisone dose equivalent of at least 1825 mg (average 5 mg/day) in the prior year; (7) indicators of hip, spine, humerus, wrist, or other clinical fractures; and (8) bone mineral density (BMD) T-score. Baseline fracture history was determined from hospitalization, institutional care, emergency or ambulatory visit diagnoses of clinical fracture within the 5 years prior to the index date (ICD-9 805, 807–815, 817–825, 827–829, excluding codes associated with open fractures, spinal cord injury, fractures of the skull, face, fingers or toes, high energy trauma (ICD-9 E800-E848), and fracture under age 40 years), classified by fracture site. For time-dependent fracture events during follow-up, we identified fractures of the hip, spine, humerus, wrist, and other clinical site based on qualifying diagnoses restricted to hospitalization, emergency, orthopedic and urgent care encounters for new fractures events occurring in the prior 12 months. BMD findings up to 5 years before BP initiation and during follow-up included femoral neck, total hip, and lumbar spine assessed by dual energy X-ray absorptiometry (Hologic, Inc.; Marlborough, MA). Accompanying T-scores were calculated using peak BMD derived from the manufacturer and from the NHANES III reference data for non-Hispanic white women according to expert recommendations [27] and were updated each quarter based on data from any new BMD test in that quarter (if available) or were otherwise carried forward. For each BMD study, the lowest T-score of the femoral neck, total hip and lumbar spine was used. Those without available BMD at baseline (27.6%) had their BMD T-score imputed based on the mean value for the cohort (T-score − 2.5), and an indicator of whether the BMD T-score was imputed was included in analyses. The most recent BMD (T-score) measurements were considered in analyses, categorized as above − 2.0, − 2.0 to − 2.4, − 2.5 to - 2.9, and ≤ − 3.0.

### Statistical analyses

**Fig. 2:**
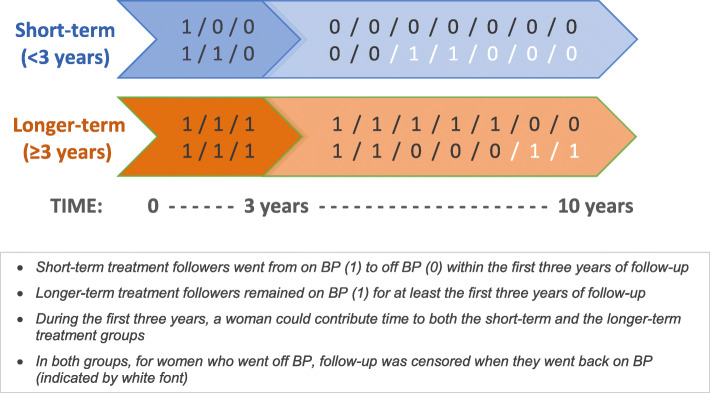
Simplified schematic showing examples of the classification of treatment group based on bisphosphonate (BP) exposure during the first ten years of treatment

We used inverse probability weighting [10, 11] to estimate the counterfactual cumulative incidence (risk) of AFF if women followed one of two stochastic [28–32] BP exposure regimens: (1) Short-term treatment: discontinuation of BP therapy within the first 3 years of BP initiation (with equal probability of BP discontinuation in each quarter of years 1–3), followed by continued absence of BP exposure based on a PDC < 50%. A woman who discontinued BP within the first 3 years of follow-up would continue to contribute follow-up time to the short-term treatment arm while off BP, but her follow-up would end if, and at the time she went back on BP. (2) Longer-term treatment: continuous exposure to BP for three or more years. The longer-term treatment arm required continuous exposure for the first 3 years, after which women could discontinue (with equal probability of BP discontinuation in each quarter of years 4–10) or remain on treatment (Fig. 2). Note that a woman could contribute person-time to both the short and longer-term treatment arms while continuously on BP treatment during the first 3 years. Our approach aimed to emulate [33] inferences from an ideal clinical trial where women would have been randomly assigned to the short- or long-term treatment arms above.

Analytic datasets were created in SAS 9.3 (Cary, NC) using the MSMstructure SAS Macro [34]. Measurements on exposure, outcome (subsequent AFF), censoring and time-dependent covariates were updated every quarter between the date of BP initiation until the end of follow-up.

We used inverse probability weighting instead of standard covariate adjustment methods in order to properly adjust for potential time-varying confounding affected by prior exposures. Time-dependent confounding occurs if some factor (e.g. BMD) is affected by past BP exposure, impacts future BP exposure decisions, and additionally affects future AFF risk. Inverse probability weighting was used to fit the stochastic-intervention analog of a saturated marginal structural model (MSM) [10] to estimate the two discrete-time, counterfactual hazard functions [35, 36] (i.e. the hazards in each of the two arms of the ideal randomized experiment we aimed to emulate). These inverse probability weighted hazard estimates were then mapped into estimates of counterfactual AFF-free survival over 10 years of follow-up, as well as estimates of the adjusted risk differences for AFF comparing the longer-term (≥3 years) to the short-term (< 3 years) BP treatment arm. We compared differences in area under the two (discrete-time) counterfactual survival curves and report corresponding *p*-values [36].

The propensity scores that define the inverse probability weights were estimated using multivariable logistic regression with all previously described covariates included. Propensity score models were additionally adjusted for (1) current follow-up time, (2) time since the most recent BMD testing and (3) indicators of whether vitamin D levels were assessed and eGFR and BMD T-scores were imputed. Separate logistic models were fit to estimate the probability of BP initiation and continuation. The model for treatment continuation at any given quarter of follow-up also included a main term for cumulative exposure to date. Four additional logistic models were separately fit to estimate the propensity scores for each of the four censoring events (death, end of study, disenrollment, and occurrence of exclusion event). All inverse probability weights were stabilized and truncated at 50 [37, 38]. The inverse probability weights were centered around 1, with median values of 0.88 (interquartile range (IQR): 0.40–1.23) and 1.05 (IQR: 0.92–1.53) for short-term and longer-term treatment followers, respectively. Truncation of stabilized weights affected less than 0.2% of the observations. Analyses were performed using the *stremr* package [39, 40] in R [41]. A *p*-value less than 0.05 was considered significant.

## Results

Among 87,820 eligible women who initiated oral BP, the mean age (± SD) was 68.6 ± 9.1 years, and 66% were non-Hispanic White, 4% Black, 10% Hispanic, and 18% Asian (Table 1). Overall, 47% were overweight or obese, 11% had a Charlson-Deyo Comorbidity Index (CCI) of 3 or more, 7% had diabetes mellitus, 3% had rheumatoid arthritis, 18% had an eGFR consistent with chronic kidney disease, and 16% had evidence of vitamin D deficiency (60% tested within 5 years). Of the 63,595 women (72%) with available BMD measurements before cohort entry, 59% had evidence of osteoporosis. One in seven women had a major osteoporotic fracture in the 5 years before BP initiation, including one in twenty-five with a hip fracture.

**Table 1:** Baseline characteristics of women who initiated bisphosphonate treatment

Characteristics	All Women (***N*** = 87,820)
Cohort entry year before 2008	47,771 (54.4%)
Age, *mean ± SD*	68.6 ± 9.1
Race/Ethnicity	
White	57,680 (65.7%)
African-American/Black	3321 (3.8%)
Hispanic/Latina	9177 (10.4%)
Asian/Pacific-Islander	15,761 (17.9%)
Other/Mixed/Unknown	1881 (2.1%)
Index Body Mass Index Category (kg/m^2^)	
Normal/Underweight (BMI < 25)	46,399 (52.8%)
Overweight (BMI 25 to < 30)	28,354 (32.3%)
Obese (BMI ≥30)	13,067 (14.9%)
Current Smoking	12,729 (14.5%)
Estimated low educational attainment based on US Census block	11,313 (12.9%)
Estimated low household income based on US Census block	5013 (5.7%)
Charlson Comorbidity Index (Deyo modification)	
0	50,993 (58.1%)
1–2	27,285 (31.1%)
≥ 3	9542 (10.9%)
History of medical conditions	
Diabetes	6544 (7.5%)
Rheumatoid arthritis	3000 (3.4%)
Grade 3 chronic kidney disease (eGFR 59–30 mL/min/1.7m^2^)	15,350 (17.5%)
Vitamin D deficiency (25OHD < 20 ng/mL)	14,459 (16.5%)
Relevant medication exposures	
Estrogen	5355 (6.1%)
Raloxifene	355 (0.4%)
Aromatase inhibitors	1792 (2.0%)
Proton pump inhibitors	10,070 (11.5%)
Glucocorticoids (prednisone equivalent 1825 mg/year)	3401 (3.9%)
Fracture history in the five years prior to bisphosphonate initiation	
Major osteoporotic fracture ^a^	12,575 (14.3%)
Any clinical fracture	23,391 (26.6%)
Bone mineral density	63,595 (72.4%)
T-Score, *median (IQR)*	−2.6 (−3.0, − 2.0)
Osteoporosis ^b^	37,420 (58.8%)
Osteopenia ^b^	23,549 (37.0%)

Numbers represent N (percent) unless otherwise indicated

^a^ Includes fractures of the hip, humerus, wrist, or spine

^b^ Osteoporosis defined as: T-score ≤ − 2.5; Osteopenia defined as: − 2.5 < T-score < − 1.0

**Fig. 3:**
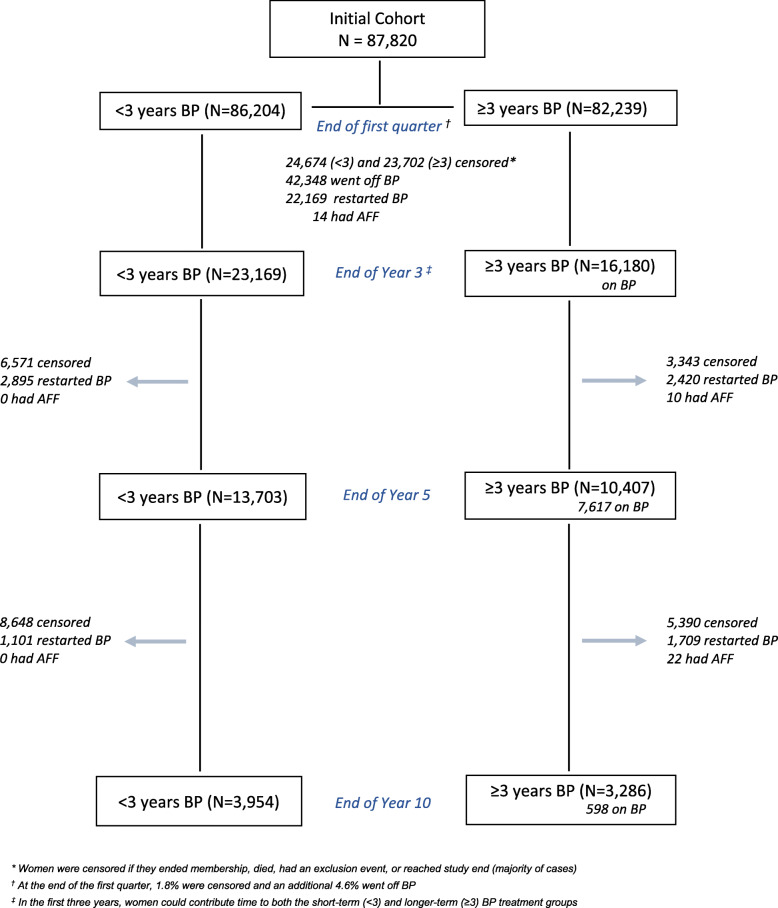
The number of women who entered and continued in the short term (< 3 years) and longer term (≥3 years) oral bisphosphonate (BP) treatment groups during ten years of follow-up

As depicted in Fig. 3, the short-term (< 3 years) treatment group included 86,204 women who entered follow-up (after 1.8% were censored in the first quarter), of whom 23,169 discontinued BP within the first 3 years (and did not restart BP and were not censored by the end of year 3); by the end of year 5 and year 10, there were 13,703 and 3954 women remaining in this arm, respectively. The longer-term (≥3 years) treatment group initially included 82,239 women who entered follow-up (after 1.8% were censored and an additional 4.6% went off BP in the first quarter), of whom 16,180 continued BP for at least three consecutive years; by the end of year 5 and year 10, there were 10,407 and 3286 women remaining in this arm, respectively. When we compared the baseline characteristics of women who remained in each group at the end of year 3 (23,169 in the < 3 years BP and 16,180 in the ≥ 3 years BP treatment group), we found that women in the longer treatment group were more likely to be Asian (21.9% vs 15.3%), to have initiated treatment before 2008, to have osteoporosis (64.0% vs 53.6%), and to have slightly lower comorbidity burden, but were less likely to have documented low vitamin D (8.8% vs 18.0%), obesity (11.0% vs 16.5%), prior fracture (21.5% vs 26.2%), or current smoking (9.8% vs 15.1%). Only small differences were seen for the remaining covariates.

A total of 46 confirmed incident AFFs occurred among women in the treatment groups during follow-up: 14 occurred in the first 3 years and 32 occurred after 3 years, the latter observed only in the longer-term treatment group (Fig. 3). All AFF cases were managed surgically with placement of an intramedullary rod.

**Fig. 4:**
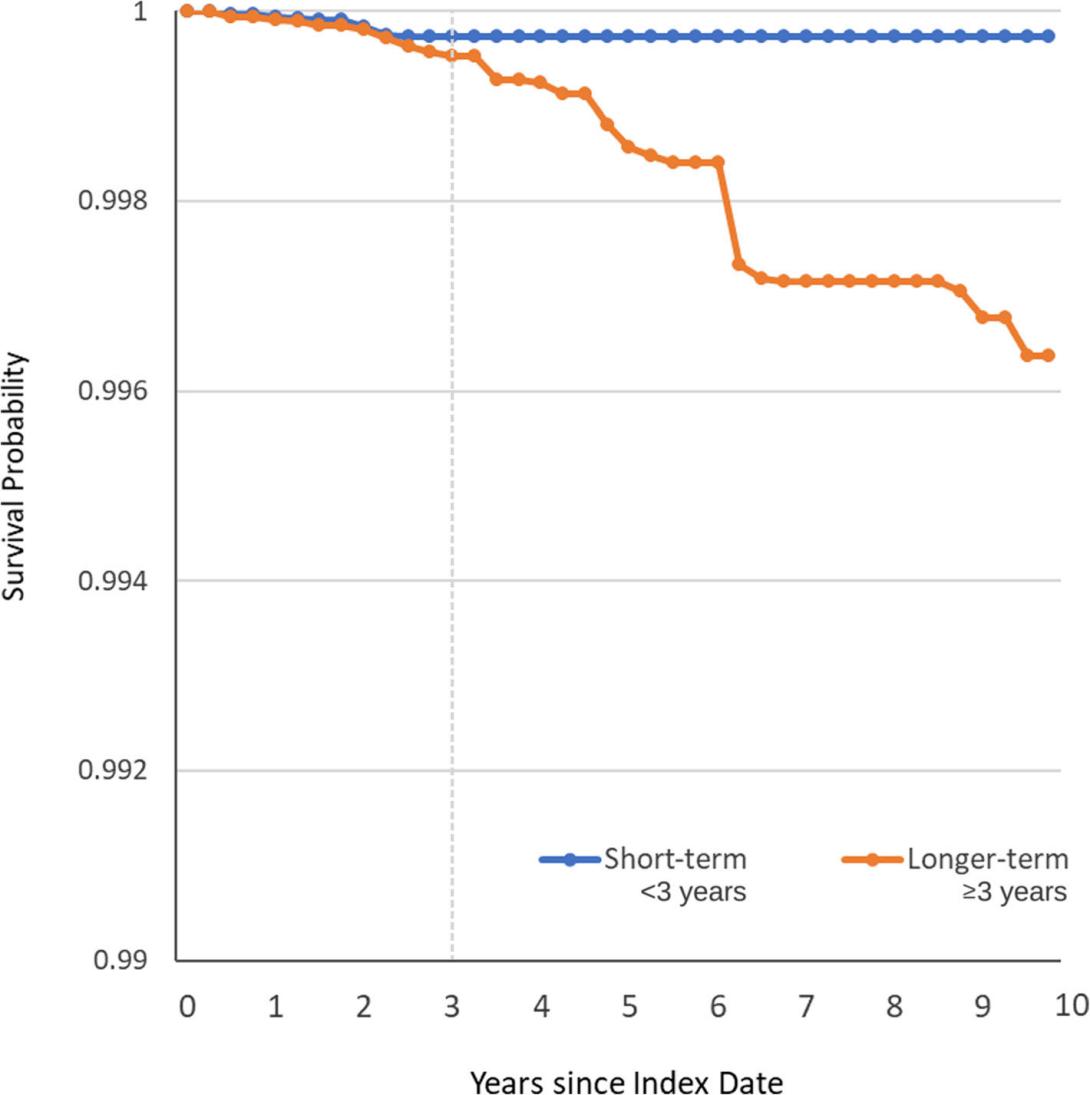
Adjusted survival curves representing time to first atypical femur fracture over ten years for women who interrupt BP in the first three years (Short-term) and those who continue BP treatment for a minimum of 3 years (Longer-term)

Adjusted survival curves for women under the short-term (< 3 years) and longer-term (≥3 years) BP treatment regimens are shown in Fig. 4. Discontinuation of BP treatment prior to 3 years resulted in greater AFF-free survival than continuation of BP treatment for three or more years, and the differences in the areas under the survival curves were statistically significant (*p* = 0.004). The survival curves appeared to separate early during follow-up, with statistically significant differences in the adjusted cumulative incidence by the end of 4 years of follow-up. The adjusted cumulative incidence of AFF was 27 per 100,000 (95% confidence interval, CI: 8–46) for < 3 years BP compared to 72 per 100,000 (95% CI: 31–113) for ≥3 years BP at the end of year 4, with an adjusted 4-year risk difference of 45 per 100,000 (95% CI: 10–80).

At the end of 5 years of follow-up, the adjusted cumulative incidence of AFF was 27 per 100,000 (95% CI: 8–46) for < 3 years BP, compared to 120 per 100,000 (95% CI: 56–183) for ≥3 years BP. The adjusted 5-year risk difference was 93 per 100,000 (95% CI: 30–160). Similarly, at the end of 10 years, the adjusted cumulative incidence was 27 (95% CI: 8–46) and 363 (95% CI: 132–593) per 100,000 for < 3 years and ≥ 3 years BP, respectively. The adjusted 10-year risk difference was 336 per 100,000 (95% CI: 110–570). Among women in the longer-term (≥3 years) treatment group, the median duration of continuous BP treatment at the end of years 5 and 10 (based on a PDC ≥50%) was 5.0 (IQR 4.7.-5.0) and 6.9 (IQR 5.0–9.0) years, respectively.

## Discussion

In this study, we found a significantly higher risk of AFF if women continued BP for 3 years or beyond compared to discontinuing BP in less than 3 years. The five-year and ten-year cumulative incidences of AFF were more than four-fold and ten-fold higher if women continued BP beyond 3 years. Although the risk of AFF if women were treated at least 3 years was low (five-year and ten-year cumulative incidence of 1 and 3 per 1000), our findings demonstrate a risk differential at a BP exposure duration threshold earlier than the five-year time point when BP treatment is typically reassessed.

Other studies have reported increases in AFF risk with longer duration of treatment, but few have examined the risk within the first 3–4 years. Dell and colleagues were the first to report a graded increase in AFF incidence with longer duration of exposure, including potential differences among women with 2–4 years of use compared to those with some but less than 2 years of use [4]. This early descriptive study focused on examining AFF incidence with increasing BP duration and did not adjust for confounders that might have affected AFF risk or the propensity to continue treatment (e.g. osteoporosis risk factors). The present analysis extends that work, as well as similar findings reported by our group [6], by showing an excess risk of AFF with treatment beyond 3 years, after controlling for potential time-dependent confounding in the causal pathway between exposure and outcome. We chose a three-year exposure threshold for our study since this represents the lower range of BP treatment duration beyond which clinical trial safety data are limited [42]. Randomized placebo-controlled trials of oral BP therapy [9] did not report an increase in risk of AFF with 3–5 years of use but were underpowered to assess this complication. Investigators in Sweden also observed trends consistent with a gradient of risk as duration increased from one to 4 years of BP, with much higher relative odds of AFF after 4–5 years of treatment [8].

Prospective randomized trials examining the potential harms of AFF with continued BP treatment would be both unethical and impossible to implement due to the required size [9]. Instead, causal inference analytic methods such as inverse probability weight estimation may be helpful to gain insights into BP-associated risks using large, real-world clinical populations. Such approaches aim to control for measured confounders, including factors such as time-updated fracture events and BMD that may be used by clinicians to determine BP continuation. While residual unmeasured confounding is possible, confounding by indication poses a greater theoretical risk for fragility fractures than for AFF. Other experts also recognize that the association of BP and AFF reported in observational studies is unlikely to be fully accounted for by unmeasured confounders [1].

Our study has some limitations. The analyses were conducted using data from clinical settings where BP regimens are typically not followed for long periods of time. Exposure was defined using PDC at 90-day intervals which may result in potential misclassification, although AFF risk is unlikely to be influenced by small variations in PDC criteria. We also did not differentiate BP dose. However, most treated patients received alendronate at a dose equivalent to 70 mg/week (10 mg/d) [43] and even half the dose (5 mg/day) is substantial and has been used in clinical trials [44–47] and for osteoporosis prevention, due to the long half-life of BP. Our study did not examine other antiresorptive osteoporosis therapies that have also been associated with AFF, including denosumab [48]. Finally, propensity scores used to adjust for factors associated with treatment continuation were estimated using parametric regression models rather than more flexible machine learning approaches which could result in residual confounding by observed covariates [36]. The strengths of our study include access to an extremely large and diverse population of women who initiated BP therapy, among whom both exposure and outcome were carefully characterized during up to 10 years of follow-up [6]. Centralized data from the electronic health record, pharmacy databases, and coded diagnoses were integrated with both radiology reports and imaging in a population with carefully defined AFF outcomes [6]. We also used analytic methods that accounted for numerous potential factors that simultaneously could be linked to treatment continuation and AFF risk.

## Conclusions

Our study extends previous findings by our group and others of the relationship between length of BP treatment and AFF risk. Importantly, we now report a significant difference in the risk of AFF after three or more years of treatment. Current guidelines suggest re-evaluation of BP continuation after 5 years [49–52]. However, our present findings suggest that AFF risk may increase even prior to 5 years and further studies are needed to guide counseling, monitoring, and long term treatment decisions in patients at risk for osteoporotic fracture. Additional studies should also address the long-term risk of usual osteoporotic fractures, such as forearm (wrist) and hip fractures.

## Data Availability

The clinical datasets generated and/or analyzed in the current study were obtained under human subjects research approval with a waiver of informed consent. As specified in the study protocol, they cannot be distributed externally. External investigators interested in these data may contact the corresponding author.

## Abbreviations

25OHD: 25-Hydroxy-vitamin D
AFF: Atypical femur fracture
ASBMR: American society for bone and mineral research
BMI: Body mass index
BP: Bisphosphonate
CCI: Charlson-deyo comorbidity index
CI: confidence interval
CKD: Chronic kidney disease
eGFR: Estimated glomerular filtration rate
ICD: International classification of diseases
KPNC: Kaiser permanente Northern California
PDC: Proportion of days covered
